# Initial community perspectives on the Health Service Extension Programme in Welkait, Ethiopia

**DOI:** 10.1186/1478-4491-5-21

**Published:** 2007-08-24

**Authors:** Haile Negusse, Eilish McAuliffe, Malcolm MacLachlan

**Affiliations:** 1 Centre for Global Health, Trinity College Dublin, Dublin 2, Republic of Ireland; 2 School of Medicine, Trinity College Dublin, Dublin 2, Republic of Ireland; 3 School of Psychology, Trinity College Dublin, Dublin 2, Republic of Ireland

## Abstract

**Background:**

The Health Service Extension Programme (HSEP) is an innovative approach to addressing the shortfall in health human resources in Ethiopia. It has developed a new cadre of Health Extension Workers (HEWs), who are charged with providing the health and hygiene promotion and some treatment services, which together constitute the bedrock of Ethiopia's community health system.

**Methods:**

This study seeks to explore the experience of the HSEP from the perspective of the community who received the service. A random sample of 60 female heads-of-household in a remote area of Tigray participated in a structured interview survey.

**Results:**

Although Health Extension Workers (HEWs) had visited them less frequently than planned, participants generally found the programme to be helpful. Despite this, their basic health knowledge was still quite poor regarding the major communicable diseases and their vectors. Participants felt the new HESP represented an improvement on previous health provision. HEWs were preferred over Traditional Birth Attendants for assistance with labour

**Conclusion:**

While the introduction of HEWs has been a positive experience for women living at the study site, the frequency of visits, extent of effectively imparted health knowledge and affects of HEWs on other health providers needs to be further explored.

## Background

While low income countries have limited resources for training healthcare professionals, the migration of those who are trained to conventional international standards has made dependence on such cadres increasingly precarious [1]. Low income countries are therefore increasingly looking to new 'mid-level cadres' (health workers who work 'above' the level of responsibility usually afforded health workers with similar training in higher income countries) to provide healthcare [2]. Research is urgently needed on the impact of these new categories of staff upon the provision of better health services in low income countries [3].

It is widely acknowledged that Ethiopia has an inadequate health service, with most of the rural, nomadic pastoralist and fringe areas having little access to it. However, even those services that do exist are underutilized due to economic and social barriers [4]. In response to Ethiopia's chronic shortfall in conventionally qualified health workers, and amidst some of the worst health indicators in Africa – including the second largest number of HIV positive people on the continent – the Ethiopian government introduced the Health Service Extension Programme (HSEP) in 2003. This innovative programme is the community-based foundation stone of the government's new primary healthcare strategy.

By 2005, 2612 HEW's had been deployed in rural villages across the country, following a one-year post-secondary school training in essential public health (child care) and hygiene (for instance, cooking practices and latrine construction), including health promotion (concerning, for instance, HIV/AIDS, Malaria and tuberculosis) and limited treatment interventions (the provision of medication to treat malaria). Although not directly comparable, as it focused only on lay health workers, a recent Cochrane review [5] supported their effectiveness in improving immunization uptake and in reducing child morbidity and mortality in low and middle-income countries. Lewin et al.'s review gives support to calls for the development of health worker cadres that have shorter training, work in the community and may have more narrowly focused skills [6-8]. The present paper is a provisional exploration of a new cadre of health workers in Ethiopia.

By mid 2006, 398 HEWs had been deployed in 33 rural villages of the Tigray Region, of Ethiopia. This study, undertaken one year after the initial implementation period, sought early feedback from the villagers who had experienced the service, in order to identify areas that might need further development as the programme evolves so as to help the programme achieve its ambitions aims. The research was conducted in six villages in Welkait, one of the most remote areas in Tigray, northern Ethiopia, and as such, one of the most challenging areas in which to improve health services. It was motivated by the need to increase understanding of human resources for health in low income countries, and in particular the impact of a new cadre of health workers. Specifically, we sought to ascertain the knowledge gained by community members through the HEW programme; the degree of community support for the programme and community perceptions regarding HEWs.

## Methods

### Context

It is important to understand the extremely challenging context in which the HEWs reported on here were operating. Adiremets is the central area of theWelkait woreda (or administrative district) in which the study was set. It is 700 km from the regional capital, Mekelle, and 1500 km from the Ethiopian capital, Addis Ababa. Over three quarters of the woreda population live more than 10 km (> 2 hours one way walking time) from any sort of health provision [9]. Almost all (96%) of the woreda's 121 572 (DHO) people live in a rural area; there are only 112 registered pit latrines; one motorised and 54 hand pumps; and 4 protected wells, giving only 18% of the population access to clean drinking water. There is one central digital public phone and few community roads (only used during the dry season) with no electric supply to the district. Malaria, intestinal parasites, diarrhoea and tuberculosis are prevalent, with malaria accounting for approximately a quarter of the disease-related burden [9].

### Sampling and procedure

A cross-sectional community-based interview survey using quota-based random sampling across 6 villages was conducted in Welkait. The interview included some open and some closed questions. It was prepared in English and translated into Tigrinya, and then back-translated into English, to ensure consistency. The themes addressed are reported below.

Six villages in the district which had received the HEW programme for a period of one year constituted the study site. All female household heads above 18 years, who were permanent residents of the study area and had been visited by HEWs for the last year, comprised the study population. Ten households from each kushet (village) were selected by simple random sampling, of every third household moving in a straight line, until the predetermined number of ten households (that fulfilled our criteria) from each village was met.

The average age of participants was 32.8 (SD = 8.23) years, 93% were illiterate, and 77% had a household income of less than $150 per year. Forty-nine women were married, 10 divorced, & 1 single. The average walking time to the nearest health facility was 3.35 hours, each way.

## Results

### Contact with Health Extension Workers

All participants had received visits from their HEW (recall this was an inclusion criteria), although only 12% had seen their HEW at the recommended weekly intervals, and 3% at two-weekly intervals. Some 85% received visits at only monthly or less frequent intervals. Some 27% did not know the name of their HEW.

### Themes addressed by Health Extension Workers

As part of the HEP, HEWs are supposed to provide education to women in households on a number of issues. Using an open question format, we asked participants what they had received education on. To summarise this data we content analysed the responses into themes. In descending order, the following themes were reported as being addressed: personal and environmental hygiene (83%), cooking practices (75%), cleaning and plastering of house (47%), construction of pit latrine and waste disposal (37%), immunisation of children (23%), separation of people and animals (22%), and use of contraception (13%). While it is possible that HEWs judge that not every household needs education on all of the issues above (perhaps because they already indicate awareness of them), it is likely that the coverage of issues may be less than optimal, as, for instance, pit latrine construction, child immunization and contraceptive use, are under-practised behaviours according to independent research (HMIS, 2005).

### Health knowledge of respondents

Participants were subsequently asked directly if the HEWs taught them about disease control and prevention, to which 80% responded positively. Those participants were asked to name the three most common communicable diseases: HIV/AIDS, Malaria and tuberculosis were named by 53%, 57% and 68% respectively of the 48 respondents; although only 18% mentioned all three. As regards understanding of how these diseases could be communicated, less than half (43%) knew that unprotected sex was a mode of transmission for HIV/AIDS, or that malaria was transmitted by mosquito bites (47%); while just over half (52%) knew that TB was an airborne disease.

### Participants' impressions of Health Extension Workers

Participants were asked to describe their own relationship with their HEW using a 4-point scale. The responses given were "very helpful" (58%), "a bit helpful" (30%), "doesn't make much difference" (3.3%) and "a bit unhelpful" (8%); Nobody endorsed the "very unhelpful" option. Participants also reported on their impression of HEWs' knowledge, again using a closed response format. They indicated that the HEWs' knowledge was "very good" (58%), "medium" (18%), and "poor" (5%), with 18% indicating that they were not able to evaluate the HEWs' knowledge.

Sixty percent of the sample indicated that HEWs helped them with home treatment of common illnesses, and all but one of these was for febrile illnesses, including malaria. We also wanted to know what effect the presence of HEWs might have on services provided by other cadres of health workers, and in particular, Traditional Birth Attendants, in this region. This was felt to be a useful point of comparison as TBAs are one of the few groups of co-existing health service providers in the region. Ninety-three per cent of participants indicated that they would prefer HEWs to assist them during labour, rather than Traditional Birth Attendants. The reasons given in discussion of this response were that the HEWs seemed more knowledgeable, but also that they were closer to hand, and/or that there were no TBAs in their village. Finally, 57% of all respondents reported that the Health Services Extension Programme offered by the HEWs represented an improvement over previous health provision.

## Discussion

The results reported here represent a preliminary review of the impact of a specific group of HEWs, working in perhaps some of the most challenging conditions to be found in Ethiopia. Yet it is in just such remote and poorly supported environments that the benefits of the HSEP may be most valuable. Following the one-year training programme, which is targeted at (only) female school leavers, the HEWs are assigned to the villages with which they are to work. Although the HEWs were supposed to visit households weekly, this had not happened. The reasons for this clearly need to be ascertained, but anecdotal accounts suggested a lack of administrative support and monitoring may be partly responsible. Indeed, it has been suggested that particularly for lower-level cadres, the provision of appropriate supervision to encourage an ethic of professionalism, is likely to be very important [3]. The strengthening of support systems for HEWs may therefore be a fruitful avenue for further investigation.

The low levels of knowledge reported by participants regarding the major communicable diseases is very worrying. Imparting health-promoting information in a non-standard environment, when mothers may have other demands being placed upon them, is without doubt a challenging task. However, the 'bringing into the home environment' of the information and the HEW is a crucial element of the HSEP, both symbolically and practically. Given the well established difficulties of communicating health information to poorly educated people [10], it may be that more attention should be paid during training to the process of imparting information and to villagers' preparedness to assimilate such information.

While personal circumstances no doubt influence the topics HEWs discuss with women, some topics – e.g. contraception & immunization – clearly need wider discussion. We would recommend the use of focus group discussions with HEWs to explore the reasons why some topics are covered in some households, but not in others; and perhaps to identify ways of enabling HEWs to more effectively advise on areas that they feel less comfortable discussing with householders.

The fact that an overwhelming majority of participants expressed a preference to be attended by a HEW rather than a Traditional Birth Attendant during labour is an interesting and rather surprising finding. Provision of a health worker with midwifery skills at every birth, coupled with access to emergency hospital obstetric care are the most crucial interventions for safe motherhood. Over the past 15 years many TBAs have been given midwifery training as part of the Safe Motherhood Strategy. The HEWs in Ethiopia do not receive training in midwifery skills, yet this research suggests that they may be called upon to attend births in the community. There has been much debate about the effectiveness of TBAs, with some claiming that they have had little impact on maternal mortality [11]. However, other studies have shown clear community preferences for TBA's health care particularly amongst rural dwellers [12]. This perception is thought to be partially influenced by the fact that community members see TBAs as members of their extended families who can understand and communicate in the local language with an understanding of the local culture. It may be that this same perception applies to the HEWs. Whilst acknowledging that there is wide variability across developing countries in the level of knowledge, skills and experience of TBAs, the unintentional undermining of their role by other cadres of health workers is a real danger. As Walraven & Weeks point out "we are in danger of wiping out the useful work along with the weaknesses, rather than building on strengths and correcting shortcomings" [11]. What is needed therefore is not another cadre of health workers (HEWs) replacing or 'competing with' TBAs, but a cadre that can complement the work of TBAs to ensure a more comprehensive coverage in terms of maternal health services. Finally, we wish to stress the importance of not drawing substantive conclusions from the small sample that participated in this research, and the corresponding small number of TBA's about whom opinions were sought. We further acknowledge that the training of TBA's can vary considerably and that in comparing HEWs with TBAs were are not comparing like with like, in terms of level of education, length of training, remuneration, etc. These issues make all the more critical the thinking through of optimal skill mix across different health providers.

HEWs do provide treatment, but only of febrile diseases (mostly malaria). The treatment of intestinal parasites and diarrhoeal diseases (as the other most prevalent diseases in Ethiopia) should be encouraged in the training of HEWs, particularly as these rural communities live such distances from the nearest health facility. Another role the HEWs could play is in TB treatment. They could address this also if they had been trained in direct observation treatment (DOTS), a treatment regime which is also being considered for anti-retroviral administration.

Despite the challenges noted above, the HEWs were broadly seen as helpful, and as improving general health service provision. It is important that the gaps in their knowledge and in particular their abilities to impart information are addressed in the training and support of HEWs. This study points to the need for locally appropriate comprehensive healthcare plans, linking and maximizing the skills of the HEWs with those of other health workers, and of providing HEWs with appropriate levels of support and supervision, perhaps especially in the early stages of their deployment.

## Conclusion

This research is limited by the fact that we cannot with certainty ascribe any changes to HEWs' inputs, because the design is neither comparative nor longitudinal. However, within these limitations, this research indicates noteworthy patterns of behaviour that may usefully be fed back into service development. Although HEWs visited village households less often than is recommended in the HSEP, most respondents had positive feeling about the service offered and felt it to be an improvement compared to before the HSEP was implemented. Despite this, respondents had worryingly poor knowledge about major communicable diseases. They also felt that HEWs were preferable to Traditional Birth Attendants for assistance with maternal care. The supervision and support of HEWs should be reviewed along with the methods used to transfer knowledge and possibly the scope of their therapeutic interventions. This preliminary investigation is of a modest scale and was set in a particularly challenging geographical area for health service provision. Nonetheless, it is hoped these results can contribute to further strengthening this innovative and much needed health provision programme.

## Competing interests

The author(s) declare that they have no competing interests.

## Authors' contributions

HN contributed to the design, data collection and analysis. EM and MM contributed to the design, analysis, and write up. All authors read and approved the final manuscript.
